# A Review of Direct Recycling Processes for Lithium-Ion Battery Cells

**DOI:** 10.3390/ma18245608

**Published:** 2025-12-13

**Authors:** Michał Łach, Agnieszka Przybek, Piotr Duda, Piotr Bielaczyc

**Affiliations:** 1 Department of Materials Engineering, Faculty of Materials Science and Physics, Cracow University of Technology, al. Jana Pawła II 37, 31-864 Kraków, Poland; michal.lach@pk.edu.pl (M.Ł.); agnieszka.przybek@pk.edu.pl (A.P.); 2 Interdisciplinary Center for Circular Economy, Cracow University of Technology, ul. Warszawska 24, 31-155 Kraków, Poland; 3 CUT Doctoral School, Cracow University of Technology, Warszawska 24, 31-155 Cracow, Poland; 4 Chair of Thermal and Process Engineering, Faculty of Mechanical Engineering, Cracow University of Technology, al. Jana Pawla II 37, 31-864 Cracow, Poland; 5 Department of Motor Vehicles, Faculty of Mechanical Engineering, Cracow University of Technology, al. Jana Pawla II 37, 31-864 Cracow, Poland; piotr.bielaczyc@pk.edu.pl; 6 BOSMAL Automotive Research & Development Institute Ltd., 43-300 Bielsko-Biala, Poland

**Keywords:** lithium-ion batteries, direct recycling, cathode regeneration, circular economy, LCA/TEA, process modeling

## Abstract

In recent years, circular economy principles have become a key paradigm in the design and evaluation of industrial processes, including recycling technologies. Direct recycling of used lithium-ion batteries is attracting particular attention, as it can significantly reduce energy consumption, reagent costs, and the carbon footprint of the entire process compared to traditional hydro- and pyrometallurgical methods. This paper provides an overview of the current state of knowledge, synthesizes contemporary methods of Li-ion battery cell recycling, and presents the most important achievements in the field of direct recycling, with particular emphasis on the regeneration and re-leaping of cathode materials, and discusses the implementation and economic premises. Key challenges and research gaps are also identified, including the need to use computational modeling (CFD/DEM, kinetic and data-driven models) to optimize the deactivation, separation, and regeneration stages. This review concludes that direct recycling has the potential to become the leading circular economy pathway for Li-ion batteries, provided that quality standardization and process modeling tools are developed in parallel.

## 1. Introduction

**Figure 1:**
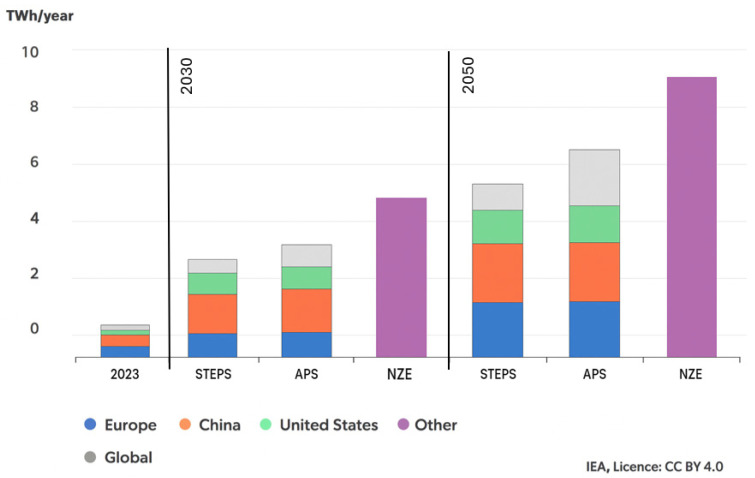
Electric vehicles’ battery demand by region, 2023–2035 [2].

Data presented by the International Energy Agency (IEA) indicate that, with the continued growth of electric vehicle (EV) sales in major markets such as China, Europe, and the United States, as well as expansion into other regions, demand for EV batteries will rise rapidly. The forecast growth is illustrated in Figure 1. In the STEPS scenario, demand for EV batteries is projected to increase 4.5-fold by 2030 and nearly 7-fold by 2035, relative to 2023. In the APS and NZE scenarios, demand is even higher, increasing five- and sevenfold by 2030 and nine- and twelvefold by 2035, respectively. For comparison, in the APS scenario, weekly EV battery demand in 2035 may reach the same level as the entire annual demand recorded in 2019 [1].

The rapid growth in the production of batteries and energy storage devices has made battery recycling an increasingly important technological and research challenge. For the emerging LIB battery recycling industry, the lack of a steady supply of end-of-life batteries remains a major economic barrier. This issue is further exacerbated in many jurisdictions where end-of-life batteries are exported to Asian countries. Currently, the main stream of LIB battery waste consists of small electrochemical cells (ECs). With the expected wave of end-of-life EV batteries in the near future, global LIB battery recycling is predicted to accelerate sharply once several supply chain gaps are bridged [3].

Recycling lithium-ion (Li-ion) batteries has become a key component of the energy transition for reasons related to raw materials, the environment, safety, and legal regulations. Li-ion batteries contain critical metals such as Li, Co, Ni, Mn, as well as graphite, the extraction of which is geographically concentrated and subject to geopolitical risk, also being highly volatile in price. Recycling enables the recovery of good quality cathode and anode materials, reducing pressure on primary deposits and stabilizing the supply chain in conditions of rapid demand growth (for EVs, energy storage) [4,5].

The environmental footprint of batteries stems mainly from the production of active materials [6,7]. Replacing some of the primary raw materials with secondary materials significantly reduces energy consumption and greenhouse gas emissions in life cycle assessment (LCA) and reduces the generation of hazardous waste [8,9,10]. Recycling also minimizes the risk of HF, solvent, and heavy metal emissions from discarded/improperly stored cells and allows for the safe neutralization of residual energy [11,12,13].

The growing stream of used batteries from the electromobility sector is forcing systemic solutions. New regulatory frameworks (e.g., European requirements for recovery levels and minimum recycled content in new batteries) make recycling not only a desirable practice, but a market requirement [3,14,15]. In addition, technological developments—from hydrometallurgy and pyrometallurgy to direct recycling with cathode regeneration—are paving the way for highly efficient material and energy recovery. From a scientific perspective, Li-ion recycling integrates the goals of raw material security, environmental impact reduction, and regulatory compliance, becoming an essential module of the circular economy for energy storage systems and EVs [16,17]. Direct recycling is a relatively inexpensive method of recycling LIBs (lithium-ion batteries), which aims to reactivate the cathode material without having to break it down into elements or chemical compounds. This approach allows for significant cost savings [18].

**Figure 2:**
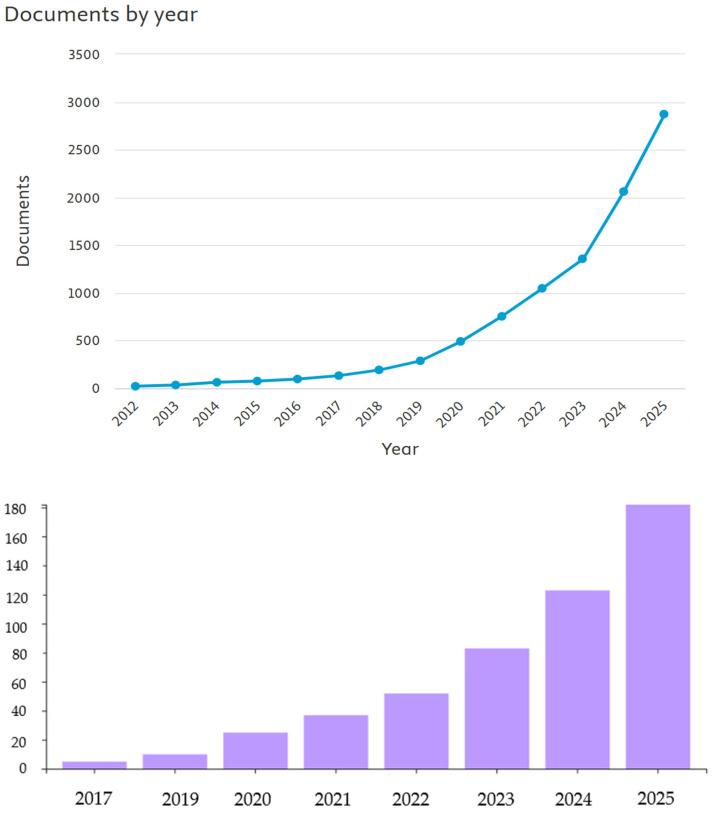
Increase in the number of scientific publications on direct recycling of Li-ion batteries (based on SCOPUS and WoS databases) [19,20].

Interest in the topic of direct recycling of lithium-ion batteries is also observed in the scientific community, as confirmed by the growing number of publications on this subject. A review of papers on direct recycling methods for lithium-ion batteries was conducted in the SCOPUS and Web of Science databases [19,20]; those databases were searched by title, abstract, and keywords fields. Figure 2 below shows how interest in this topic has developed over the years, as reflected in the increase in the number of scientific publications.

**Figure 3:**
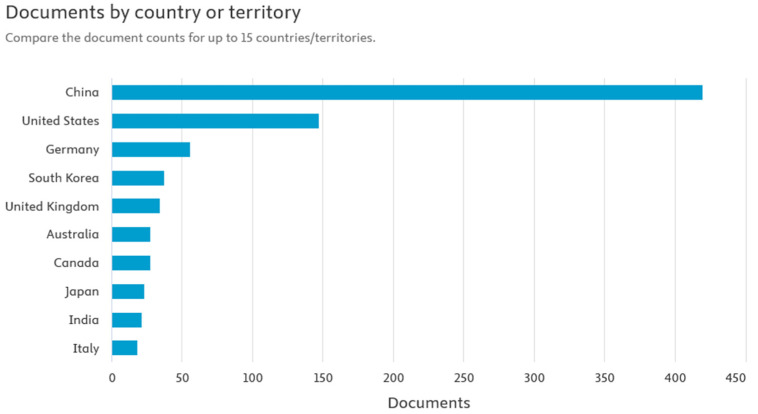
Publications related to the topic of direct recycling of LIBs in selected countries [19].

Figure 3 shows the publication activity related to the direct recycling of lithium-ion batteries (LIBs) in individual countries. The analysis of the data clearly shows that China generates the largest number of scientific publications in this area. This is entirely justified, given the country’s dominant position in the global lithium-ion battery supply chain. It is in China that most of the plants producing both cells and complete battery systems are located, as well as extensive research and development (R&D) facilities related to cathode, anode, and electrolyte technologies. China’s dominance in scientific publications may also be linked to the growing emphasis by the authorities on implementing circular economy solutions and the dynamic development of the electromobility industry. The large scale of battery production and consumption in this country generates huge amounts of used LIBs, which naturally stimulates research into their recovery and reuse. By comparison, countries such as the United States, South Korea, Germany, and the United Kingdom show significantly less, though still significant, activity in the field of direct LIB recycling research. These countries are conducting highly advanced technological work, often focused on optimizing cathode material regeneration processes and increasing the efficiency and safety of industrial raw material recovery. China’s observed advantage in the number of scientific publications not only reflects its production potential, but also indicates the strategic focus of national research policy on the development of sustainable recycling technologies, which may be a key element of the global lithium-ion battery market in the future [19].

This review describes the current state of knowledge on the direct recycling of lithium-ion batteries (LIBs) and presents the evolution of this approach in the context of the development of active material recovery technologies. The most important research directions are discussed, with particular emphasis on innovative methods for the regeneration of cathode and anode materials, and key challenges associated with their reuse in new cells are identified. The advantages of direct recycling compared to traditional hydrometallurgical and pyrometallurgical methods are presented, such as lower energy consumption, reduced pollutant emissions, and the possibility of preserving the structure and electrochemical properties of active materials. The growing importance of computer modeling and numerical simulation processes in optimizing direct recycling stages was also emphasized. These techniques enable, among other things, the prediction of material behavior during regeneration, the assessment of the impact of process parameters on their crystal structure and electrochemical performance, and the design of new, more effective recovery pathways. Computer modeling is now an indispensable tool in LIB recycling research, allowing for the integration of experimental knowledge with theoretical analysis and accelerating the development of technologies towards their commercial application. The entire study aims to identify current research trends, innovation potential, and future prospects for the development of direct recycling technologies that can significantly contribute to increasing the sustainability of the life cycle of lithium-ion batteries.

## 2. Traditional Recycling Methods

The importance of recycling lithium-ion batteries within a circular economy is increasingly recognized as a key strategy for conserving resources, minimizing environmental impact, and promoting sustainable development. With demand for lithium-ion batteries growing—driven primarily by the electric vehicle (EV) market and renewable energy solutions—it is crucial to manage these batteries effectively at the end of their life cycle. Recycling lithium-ion batteries is a key step toward establishing a circular economy that emphasizes the sustainable use of resources by ensuring that materials are reused, recovered, and reintegrated into production cycles at the end of a product’s life cycle. Recycling methods focus on recovering valuable metals such as lithium, cobalt, and nickel while also aiming to reduce the environmental risks associated with improper disposal. Ibarra et al. demonstrate that techniques using supercritical fluids to recover cobalt can achieve recovery rates of up to 99%, revealing their potential for efficient recycling, which is essential in the context of a circular economy [21]. In turn, Coyle et al. discuss the fundamental challenges and opportunities associated with recycling electric vehicle batteries, emphasizing that the implementation of effective recycling methods can significantly contribute to achieving a circular economy for these batteries [22].

Technological advances in recycling processes are crucial to realizing the benefits of a circular economy. Mohr et al. present both existing and potential future pathways for lithium-ion battery recycling, highlighting the importance of innovation and improvement in recycling processes to meet growing demand while taking into account resource constraints [23]. Furthermore, Khawula and Palaniyandy present a sustainable method for recycling lithium iron phosphate batteries, showing that technologies such as hydrothermal synthesis can facilitate circular recycling in line with circular economy principles [24]. Research has also shown that the use of efficient recycling technologies can reduce dependence on primary raw materials and lower the associated costs [25].

The economic, environmental, and technical aspects of battery recycling further underscore its importance. Filomeno and Feraco argue that recycling can alleviate market pressures by moderating raw material prices and reducing the environmental impact associated with extraction and disposal [26]. This view is supported by Meegoda et al., who suggest that a transition to a circular economy for lithium-ion batteries could significantly reduce social and environmental costs while ensuring efficient resource use and waste management [25].

Furthermore, responsibility for recycling lithium-ion batteries has implications for the regulatory framework. With the introduction of new battery recycling regulations in various regions, there is a growing need to develop structured recycling methods that are consistent with the principles of the circular economy. Neumann et al. argue that as the lithium-ion market grows, it is essential to improve recycling technologies and frameworks [17]. Yang et al. emphasize the importance of ensuring sustainability and safety in recycling processes, positioning such practices as essential elements of a sustainable energy transition [27].

Recycling lithium-ion batteries is crucial to supporting the circular economy. It helps recover valuable materials, minimizes environmental damage, and addresses the challenges associated with the growing popularity and disposal of such batteries. Continued advances in recycling technologies and practices will play a key role in achieving a resilient and sustainable battery life cycle. The recycling of lithium-ion batteries (LIBs) involves traditional methods such as pyrometallurgy and hydrometallurgy, which are crucial for recovering valuable materials while addressing environmental concerns. Both techniques have their strengths and weaknesses, and understanding their processes and implications is crucial for developing recycling strategies within a circular economy model.

Pyrometallurgy is one of the oldest methods used to recycle batteries and involves treating batteries at high temperatures to extract metals. This method is particularly effective in recovering cobalt, nickel, and copper, which are found in significant concentrations in LIB batteries [28]. Pyrometallurgy typically involves the dismantling, shredding, and thermal treatment of battery materials, ultimately resulting in the production of a metallic alloy or “black mass” that can be further refined [29]. It is worth noting that pyrometallurgical processes require high energy inputs, raising concerns about their overall environmental impact, including greenhouse gas emissions and energy consumption [30].

Hydrometallurgy, on the other hand, uses aqueous solutions to extract metals from used batteries. This method typically involves leaching processes, which use acids or bases to dissolve metals, followed by separation and purification [31]. The hydrometallurgical approach is advantageous because it allows for the selective recovery of specific metals from battery waste, which can result in higher recovery rates compared to pyrometallurgy [32]. However, hydrometallurgical processes can also cause environmental pollution if not properly managed, mainly through the generation of hazardous wastewater during the leaching stages [31]. For example, the use of large amounts of sulfuric acid in the leaching process can pose a risk if not effectively controlled [33].

In terms of efficiency, both methods have been critically evaluated. Although pyrometallurgy allows high metal recovery rates to be achieved, its significant disadvantages are considerable energy consumption and potential toxic emissions [30]. On the other hand, hydrometallurgy often involves long processing times and can be difficult to use for the selective recovery of lithium, which is crucial given the growing demand for this raw material [34]. Furthermore, a serious problem is that traditional leaching processes are often not optimized for lithium recovery, leading to losses of precious metals [35]. In order to increase lithium recovery efficiency while minimizing environmental impact, innovative solutions such as combining advanced oxidation processes with hydrometallurgy have been explored [35]. Both methods are integral to solving the problem of managing spent lithium-ion batteries. Xiao et al. emphasize the importance of developing efficient and environmentally sustainable practices that aim to improve the current shortcomings of both pyrometallurgical and hydrometallurgical methods [36]. Overall, although traditional recycling methods have become an essential means of managing spent batteries, there is an urgent need for further research and development to increase their efficiency, reduce their environmental impact, and integrate innovative technologies such as direct recycling as complementary solutions [30].

Recycling lithium-ion batteries using traditional methods such as pyrometallurgy and hydrometallurgy reflects the complexity and necessity of recovering valuable materials while ensuring compliance with environmental regulations. The continued development of hybrid approaches that combine the advantages of these traditional methodologies is critical to advancing battery recycling within a sustainable circular economy.

### 2.1. Pyrometallurgical Recycling Methods

The pyrometallurgical process usually begins with the dismantling and pre-processing of used lithium-ion batteries, in which discharged cells are shredded and cleaned to facilitate metal recovery [37]. The battery materials are then thermally treated at temperatures ranging from 1000 °C to 1500 °C. At these elevated temperatures, the organic components of the batteries are burned off, effectively cleaning the system of unwanted materials. The thermal treatment then allows the metals to be separated, which solidify into various forms of alloys, including those containing cobalt, nickel, and copper [38]. The high-temperature melting process allows for the formation of immiscible molten layers in which the metal phases can be separated from the slag [39,40].

Despite its effectiveness, the pyrometallurgical approach is not without its challenges. A significant drawback is the inherent loss of lithium during the smelting phase, as lithium typically exhibits a high affinity for oxygen and can become trapped in the slag [41]. This loss requires further hydrometallurgical processing to recover lithium from the materials left over after pyrometallurgical treatment [42]. Furthermore, the energy-intensive nature of pyrometallurgy raises concerns about greenhouse gas emissions and overall sustainability, highlighting the need for innovation or combined processes integrating hydrometallurgy to improve metal recovery efficiency [40,43].

The literature indicates that improvements can be made to pyrometallurgical refining processes to increase lithium recovery and reduce energy consumption. For example, some studies propose the development of more advanced reactor designs and the implementation of innovative reducing agents that can optimize operating parameters, ultimately leading to improved metal extraction [41,44]. In addition, combining pyrometallurgical and hydrometallurgical techniques may provide a more comprehensive solution, ensuring higher resource recovery while minimizing waste [40,45]. Pyrometallurgical recycling is considered versatile and suitable for various types of batteries, especially those with high metal content, such as nickel and cobalt [46]. Studies have shown that investments in technologies that facilitate cleaner and more efficient pyrometallurgical methods are crucial to improving the sustainability of battery recycling practices [47]. For example, integrating processes that reduce slagging and improve lithium extraction can significantly improve the overall efficiency of pyrometallurgical recycling processes [41,43].

**Figure 4:**
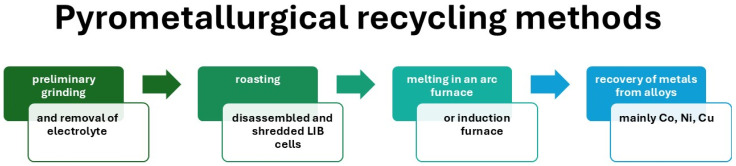
Schematic diagram of the pyrometallurgical recycling process for lithium-ion batteries (LIB), including typical stages: preliminary preparation of cells, thermal roasting, high-temperature melting, and metal recovery from the alloy.

Pyrometallurgical recycling methods are essential for recovering valuable metals from used lithium-ion batteries, facilitating their return to raw materials used in the production of new batteries. Although there are inherent challenges, such as energy consumption and lithium loss, ongoing research and technological advances are pointing toward improved efficiency and reduced environmental impact, supporting a more sustainable approach to battery life cycle management. Figure 4 shows a diagram of the pyrometallurgical recycling process for lithium-ion batteries (LIBs), including its typical technological stages. The process begins with the preliminary preparation of used cells, including their discharge, disassembly, and shredding, followed by thermal roasting to remove the electrolyte and decompose organic substances and polymer binders. The next stage is high-temperature smelting, during which metal oxides are reduced and an alloy containing valuable metals (mainly Co, Ni, Cu) is formed. In the final stage of the process, metals are recovered from the alloy obtained and slag, a by-product containing, among others, lithium and aluminum, is separated. This diagram therefore illustrates the classic sequence of steps characteristic of the pyrometallurgical approach to LIB recycling, which is dominated by high-temperature processes leading to metal recovery with the simultaneous loss of the structure of the active materials.

Pyrometallurgical methods of recycling lithium-ion batteries are among the oldest and most technologically advanced industrial solutions. This process is based on high-temperature processing of used cells, including roasting, melting, and reduction of metal oxides to obtain a metal alloy containing valuable elements such as cobalt, nickel, copper, and iron [30,48,49].

The main advantages of pyrometallurgy include technological and operational simplicity and the ability to process mixtures of different types of cells without the need for segregation. In addition, the process is highly scalable industrially and can be integrated into existing metallurgical installations, making it attractive to large metallurgical companies [50]. At the same time, this technology is characterized by significant energy consumption due to the need to conduct the process at temperatures ranging from 1000 to 1500 °C, which translates into a high carbon footprint [51]. During melting, process gases such as HF, CO_2_, and SO_2_ are produced, which require appropriate treatment [33]. In addition, lithium and aluminum in most cases remain in the slag, which limits their recovery [52]. However, the most serious limitation of pyrometallurgy is the loss of the structure of cathode materials, which prevents their direct regeneration and reuse [53].

There are several commercial installations around the world that use this method, including Umicore (Belgium), Glencore (Canada), and Sumitomo Metal Mining (Japan), which have been developing processes for recovering metals from battery waste based on metallurgical solutions for many years [54,55,56]. Table 1 summarizes the main advantages and disadvantages of petrometallurgical recycling of lithium-ion batteries (LIB) and provides examples of industrial technologies using this approach. In this process, metals are recovered in a high-temperature environment using organic solvents or hydrocarbons, which allows for the selective separation of cobalt, nickel, copper, and other metals.

### 2.2. Hydrometallurgical Recycling Methods

The hydrometallurgical recycling process typically begins with mechanical processing of used LIB batteries, which involves disassembly, shredding, and removal of any non-metallic components. The cathode materials—often consisting of lithium transition metal oxides—are then leached in acid solutions, typically using sulfuric acid (H_2_SO_4_) or hydrochloric acid (HCl) [57]. These acids effectively dissolve valuable metals such as nickel (Ni), cobalt (Co), and manganese (Mn), breaking down the solid matrix of cathode materials into ionic form [57,58]. The choice of acid and leaching conditions has a significant impact on metal recovery efficiency. For example, Ma et al. show that strong acids such as H_2_SO_4_ lead to almost complete dissolution of transition metals, in contrast to weaker acids, which result in lower leaching efficiency due to side reactions forming insoluble precipitates [58]. This highlights the need for careful selection of reagents to optimize recovery rates during the leaching phase. After dissolving metals in solution, selective precipitation is used to recover them efficiently. Precipitation often requires adjusting the pH of the leach to induce the formation of solid metal compounds, which can then be filtered out. Research suggests that various leaching and precipitation techniques, including the use of organic acids, can increase recovery efficiency and reduce waste product formation compared to traditional inorganic acids [59,60]. For example, Bae and Ryoo demonstrate increased recovery of valuable materials using organic acids as leaching agents, suggesting that these alternatives provide a less toxic and more environmentally friendly recycling pathway [60].

To further improve this process, a multi-step approach based on oxidation and precipitation can be used, as described by Ma et al., which increases the selective recovery of multiple metals from spent lithium-ion batteries simultaneously [58]. This method not only improves resource recovery rates, but also mitigates the environmental impact typically associated with traditional hydrometallurgical processes, which generate significant amounts of wastewater [61,62]. In fact, the introduction of innovative hydrometallurgical processes using both acids and more sustainable leaching agents is a key area of interest in ongoing research [62]. Despite these advances, challenges remain in improving the selectivity and efficiency of metal recovery. For example, Li et al. highlight the importance of optimizing leaching conditions and subsequent material separation techniques, which are critical for ensuring efficient recovery of lithium along with nickel and cobalt [63]. Furthermore, the environmental impact associated with the disposal of acidic wastewater requires careful management or treatment prior to release into the environment [64,65].

**Figure 5:**
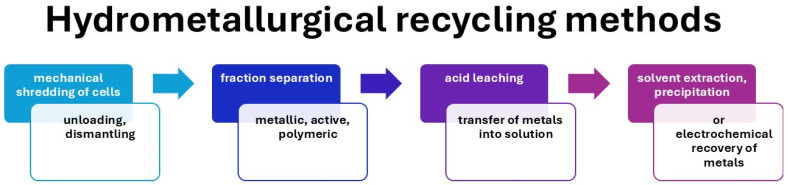
Schematic diagram of hydrometallurgical recycling of lithium-ion batteries, including mechanical shredding of cells, separation of fractions, acid leaching, and metal recovery through extraction, precipitation, or electrochemical methods.

Hydrometallurgical methods offer significant advantages for lithium-ion battery recycling, particularly due to their ability to selectively dissolve and precipitate valuable metals while minimizing waste. Although this process is promising, continuous improvements in reagent selection, process optimization, and environmental management are necessary to increase the efficiency and sustainability of these recycling methods in the context of a circular economy. Figure 5 shows a diagram of the hydrometallurgical recycling process for lithium-ion batteries (LIBs), including its typical technological stages. The process begins with the preliminary preparation of used cells, including their discharge, disassembly, and mechanical shredding, followed by the separation of the metallic, active, and polymer fractions. The next stage is acid leaching, which allows the metals to pass into solution. The resulting solution is then subjected to solvent extraction, precipitation, or electrochemical metal recovery, allowing for the selective recovery of cobalt, nickel, lithium, and other valuable elements. This diagram illustrates the classic sequence of steps characteristic of the hydrometallurgical approach to LIB recycling, which is dominated by chemical processes leading to metal recovery while maintaining the structure of active materials in solution form.

Hydrometallurgical methods of recycling lithium-ion batteries are currently one of the most frequently researched and used technologies for recovering secondary raw materials. This process is based on the chemical dissolution of cathode materials in mineral acid solutions and the selective recovery of metals by precipitation, solvent extraction, or electrolysis. Compared to pyrometallurgical methods, hydrometallurgy is characterized by lower energy consumption and higher metal recovery efficiency, including the possibility of lithium regeneration, which is usually lost in slag in high-temperature processes [48,49,66,67]. Nevertheless, these methods require the use of strong chemical reagents, which generates significant amounts of liquid waste that requires appropriate treatment before disposal [33,68]. In addition, the dissolution of active materials leads to the loss of their original crystal structure, which precludes the direct use of recovered compounds in new cells [53,69].

Despite these limitations, hydrometallurgy is currently the most technologically advanced and commercially available method of LIB recycling, used by companies such as Retriev Technologies, OnTo Technology, Fortum, and Toxco [70,71,72]. In many cases, it is also part of integrated hybrid processes combining roasting and leaching stages, which improves efficiency and reduces the environmental impact of the entire process. Table 2 summarizes the main advantages and disadvantages of hydrometallurgical recycling of lithium-ion batteries (LIBs), along with examples of industrial technologies using this approach. In these methods, the metal recovery process is carried out in a liquid environment, using acidic or organic solutions that enable selective leaching and extraction of individual elements.

### 2.3. Hybrid Methods (Pyro-Hydro)

One common approach in hybrid recycling is the pre-roasting of used LIB batteries, which serves as a preliminary thermal treatment. This pre-treatment effectively removes organic components and helps concentrate valuable metals such as nickel, cobalt, and lithium into a form that is more accessible for subsequent leaching [73]. After this stage, the thermally treated material is subjected to acid leaching using solutions such as sulfuric acid (H_2_SO_4_) or hydrochloric acid (HCl) to dissolve the metals in a liquid environment [46]. This combined process allows for high recovery rates and more efficient separation of valuable metals compared to methods used alone.

The effectiveness of hybrid processes has been highlighted in studies showing that combining pyrometallurgical methods, such as smelting, followed by hydrometallurgical refining, significantly improves overall recovery [26,74]. This two-step approach allows alloys and slag to be produced in the first stage of pyrometallurgy, which can then be selectively subjected to hydrometallurgical treatment to separate and purify lithium from other metals, thus optimizing resource utilization [74]. Furthermore, recent research on refining techniques has shown that introducing materials such as sulfuric acid into dry slag digestion can reduce silicon leaching, which is one of the main challenges in lithium recovery during hydrometallurgical processing. This method shows potential for increasing lithium recovery efficiency through innovative approaches within hybrid structures [74]. Additionally, the use of solvent extraction techniques further aids in the selective recovery of lithium and other valuable metals, offering more efficient alternatives in hybrid recycling [75]. Research indicates that combining these methods can benefit the environment by reducing the carbon footprint associated with the recycling processes themselves. By optimizing operations and reducing waste associated with both pyrometallurgy and hydrometallurgy, hybrid methods offer more sustainable solutions [46,76].

In recent years, lithium-ion battery (LIB) recycling has increasingly focused on hybrid methods that combine pyrometallurgical and hydrometallurgical processes. These hybrid approaches aim to improve recovery efficiency and reduce emissions associated with recycling valuable materials from spent batteries. Combining these processes allows the advantages of each method to be exploited, eliminating their individual limitations and increasing the overall sustainability of battery recycling.

The hybrid method typically begins with pyrometallurgical treatment, in which spent LIB batteries are pre-roasted or smelted at high temperatures. This thermal treatment effectively burns off organic components and concentrates valuable metals into alloys, facilitating subsequent material recovery. For example, Li et al. demonstrated an SiCl_4_-assisted roasting method that significantly increases the leaching rate of cobalt and lithium; temperatures between 300 and 400 °C yielded leaching rates between 66.6% for Co and 91.3% for Li [77]. This pre-treatment step optimizes the material by reducing the amount of impurities and improving the availability of metals for further hydrometallurgical processing. After this pyrometallurgical stage, the leached metals undergo hydrometallurgical processes. In this case, acids such as sulfuric acid (H_2_SO_4_) or hydrochloric acid (HCl) are used to dissolve the metals, allowing for their selective precipitation [78]. This selective precipitation of metals is a key advantage of hydrometallurgy, enabling the recovery of nickel, cobalt, and lithium with greater efficiency. Hybrid processes facilitate this by first enriching the metal content and then refining it, which improves the overall quality and quantity of the recovered materials [79].

Despite the advantages of these hybrid methods, conventional methods face problems such as high energy consumption and greenhouse gas emissions. Recent studies suggest that optimizing pyrometallurgical conditions and integrating advanced hydrometallurgical techniques not only improves recovery efficiency but also makes these processes more environmentally friendly [46]. For example, the use of ammonium sulfate roasting has been investigated as a more efficient method of lithium recovery compared to some conventional methods, reducing energy requirements and emissions [80].

Combining processes in a hybrid format can significantly reduce the carbon footprint associated with battery recycling. Dunn et al. emphasized that while traditional pyrometallurgical methods can generate higher carbon dioxide emissions, hybrid approaches significantly reduce these emissions by improving efficiency and reducing reliance on high-temperature operations [76]. Furthermore, studies indicate that integrating these methods can result in reduced energy consumption and lower greenhouse gas emissions compared to each process used individually [46]. Life cycle assessments of hybrid recycling methods consistently point to their potential for achieving greater sustainability in battery recycling. For example, Zhang et al. emphasize that combining low-temperature processes with traditional high-temperature methods can significantly reduce CO_2_ emissions associated with battery material recovery [81]. Furthermore, advances in direct recycling techniques, which can combine the characteristics of pyrometallurgical and hydrometallurgical processes, offer additional environmental and economic benefits, making them an attractive option for industry players seeking to increase sustainability [82].

The growing interest in hybrid pyro-hydro recycling methods for lithium-ion batteries reflects a significant shift toward more efficient and sustainable practices in the industry. These methods improve the recovery rate of valuable materials while reducing the environmental impact commonly associated with traditional recycling methods. As demand for lithium-ion batteries increases, the adoption of hybrid processing techniques will be critical to meeting future challenges related to resource scarcity and environmental protection.

The hybrid pyro-hydro method usually begins with a pyrometallurgical stage, in which used batteries are treated at high temperatures (approximately 1000 °C to 1500 °C). This initial phase serves to concentrate and separate metal components such as nickel and cobalt, often resulting in alloys, while the organic materials in the batteries are burned off [46,83]. After this thermal treatment, the metal-rich residues are processed using hydrometallurgical techniques, where acid leaching further separates valuable metals, such as lithium, into a leach solution [76]. An example of this approach is the technology developed by Li-Cycle, which effectively integrates pyrometallurgical and hydrometallurgical processes to recover metals, including lithium, nickel, and cobalt, from used batteries. Li-Cycle’s methodology shows the potential for high recovery efficiency while minimizing waste and emissions [82]. Similarly, Fortum’s technology uses a hybrid model, employing innovative thermal treatment followed by hydrometallurgical recovery, improving the purification and separation of valuable materials from battery waste [84]. The combination of preliminary pyrometallurgical processing with subsequent hydrometallurgical recovery significantly increases the overall metal recovery efficiency. Studies indicate that this method allows for significant recovery of key metals such as lithium and cobalt, and in some configurations, the recovery efficiency often exceeds 90% [85].

Hybrid methods can contribute to reducing greenhouse gas emissions compared to traditional pyrometallurgical practices. For example, hydrometallurgical processes, although requiring careful management, typically have reduced emissions compared to high-temperature pyrometallurgical operations [37,76]. The carbon footprint of the pyro-hydro approach can be reduced because preliminary pyrometallurgical treatment allows for the recovery of energy that would otherwise need to be obtained from external sources [46]. The hybrid approach maximizes resource efficiency while minimizing harmful by-products. By integrating these methods, it is possible to improve battery waste management, enabling the recovery of various metals with minimal waste production [82,86]. In addition, advances in solvents and biometallurgical techniques are increasing the environmental friendliness of these hybrid recovery processes [87].

The growing field of lithium-ion battery recycling highlights the importance of hybrid techniques that combine pyrometallurgical and hydrometallurgical methodologies. Innovations from companies such as Fortum and Li-Cycle exemplify the effectiveness of these integrated approaches, contributing to increased recovery rates and reduced emissions. As the demand for sustainable battery management grows, the development and optimization of hybrid recycling technologies will be crucial to closing the material loop in battery production and consumption, ultimately promoting sustainability within a circular economy.

### 2.4. Limitations of Traditional Methods

A significant drawback of traditional recycling methods is their tendency to compromise the integrity of active materials in battery cells. Conventional processes often involve high-temperature pyrometallurgical smelting, which can degrade these materials, rendering them unsuitable for direct reuse in new batteries. As a result, valuable components such as lithium cobalt oxide and other active materials may undergo irreversible changes during processing, leading to a loss of their functional properties [88]. As a result, instead of remanufacturing batteries using these materials, companies must synthesize new components, leading to increased resource consumption and waste generation.

Traditional battery recycling techniques also have a high carbon footprint and significant energy costs. For example, energy-intensive pyrometallurgical processes often require intensive heating, which contributes to greenhouse gas emissions [29]. This energy demand poses an economic challenge and undermines the sustainability goals associated with battery recycling. Researchers have pointed out that pyrometallurgy can result in higher CO_2_ emissions compared to hydrometallurgical processes, further complicating efforts to mitigate climate change [17]. The financial burdens associated with energy consumption may reduce the economic viability of recycling, especially if energy costs continue to rise.

In addition, treating wastewater and emissions from traditional battery recycling processes is complex and requires significant investment in pollution control technologies. During the pyrometallurgical phase, the generation of toxic fumes and the potential release of hazardous materials pose serious environmental and health risks, requiring strict regulatory compliance and costly treatment systems [89]. The need for robust wastewater treatment systems increases operating costs, complicates recovery processes, and often results in secondary waste streams that require further management.

Finally, traditional recycling methods focus mainly on recovering economically attractive elements such as nickel and cobalt. This approach leads to the neglect of lithium and other materials of lesser economic value, which can result in inefficient use of resources and threaten the sustainability of battery recycling [90]. The lack of comprehensive recovery strategies often results in failure to recover the essential components needed for a truly circular economy in battery production. As a consequence, essential materials may be lost, exacerbating supply shortages and increasing demand for newly mined resources [91].

Although traditional battery recycling methods play a key role in battery waste management, they have many limitations that hinder their effectiveness in promoting sustainable development. Loss of active material integrity, high carbon emissions, complex wastewater treatment, and a narrow focus on economically viable components collectively undermine efforts to support a circular economy for lithium-ion batteries. Addressing these limitations is essential for developing more sustainable and efficient recycling strategies in the future.

### 2.5. Advantages and Conditions for the Application of Pyrometallurgical, Hydrometallurgical, and Hybrid Methods

Analysis of the three main classes of lithium-ion battery recycling methods—pyrometallurgical, hydrometallurgical, and hybrid—makes it possible to determine precise areas of their optimal application depending on the characteristics of the input stream, recovery requirements, and available technological resources [3,12,17]. Differences in their efficiency result both from varying process mechanisms and from the specifics of the materials being recycled, which can display significant variability in terms of cathode chemistry, degree of degradation, or the presence of inert components [17,22,30].

Pyrometallurgical methods demonstrate the highest resistance to inconsistencies and impurities in the input stream, which makes them particularly useful in situations where entire modules or packs are sent for recycling, often without disassembly and in varying technical condition [38,39,40]. High-temperature smelting allows for quick and stable recovery processes, in which transition metals (Ni, Co, Cu) pass into the alloy, while the remaining components go to the slag or decompose. As a result, pyrometallurgy is especially effective as a “first contact” technology for streams that are not economically viable to prepare or sort, such as when processing accident-damaged, heavily degraded, or highly contaminated batteries [41,42,74].

Another significant advantage is the easy scalability of the process—metallurgical installations can operate continuously, and their capacity can be increased at relatively low modernization costs. Under industrial conditions, pyrometallurgy is also relatively insensitive to fluctuations in cathode chemistry, which can hinder recovery in wet processes. Its use is therefore the most justified choice where the waste stream is large, dynamically changing, and difficult to classify [38,39,40].

At the same time, pyrometallurgical methods have inherent limitations—the relatively low efficiency of lithium recovery, as most ends up in the slag, and the high energy demand make them less than optimal where full closure of the critical raw materials loop or minimizing the environmental impact of the process is a priority [41,42]. Therefore, their profitability is highest in locations with existing smelting infrastructure and lowest where new installations would have to be built from scratch.

Hydrometallurgical methods provide the highest selectivity of recovery and the ability to obtain compounds of very high purity, such as sulfates, carbonates, or metal hydroxides [32,33,35]. Their advantage lies in the possibility of precisely controlling leaching conditions, which enables the practically complete recovery of Ni, Co, Mn, and Li, particularly important in the context of building a circular economy for critical elements [57,59,60]. Wet processes work best in situations where the input material is properly prepared: crushed, stripped of casings, separators, and metallic components, and the chemical composition of the active fraction is known and relatively homogeneous.

Hydrometallurgy is therefore recommended where disassembly and feed preparation are possible, and the priority is to obtain final products with the highest market value and the quality required by the cathode materials industry [62,63,71]. Another advantage is its lower energy consumption compared to thermal methods, which makes it suitable for use in regions with high energy costs or strict CO_2_ emission standards [58,69,84].

However, effective application of hydrometallurgical technologies requires access to infrastructure that allows for the management of process solutions, reagents, and by-products. Due to the necessity of generating and treating process wastewater, these methods are least advantageous in locations lacking chemical treatment facilities or where liquid waste disposal is expensive or subject to environmental restrictions [33,58,71].

Hybrid methods (pyro–hydro) combine the advantages of both previous approaches, allowing for moderate roasting and selective leaching to be carried out within a single technological process [40,83]. Their advantage comes from the fact that the thermal step removes organic substances (e.g., electrolyte, PVDF), stabilizes the active material, and reduces the risk of reactivity, which significantly improves the efficiency of subsequent leaching. As a result, hybrid methods can handle streams with a greater degree of diversity than traditional hydrometallurgy while also achieving higher lithium recovery rates than pyrometallurgy [40,83,88].

These technologies are particularly cost-effective when a facility processes large quantities of diverse waste but aims for a high recovery rate of all metals, not just transition metals. In such conditions, hybridization lowers operational costs, reduces energy consumption, and at the same time simplifies further chemical processing by removing the organic fraction during the thermal stage.

However, the effective use of hybrid methods requires investment in complex infrastructure that integrates both roasting installations and wet process systems. Therefore, the profitability of such solutions increases with scale—in large plants, they achieve the best compromise between resistance to feedstock variability and efficiency in recovering all critical elements [83,88].

The optimal choice of method depends on the context: pyrometallurgy works best for large, unsorted streams; hydrometallurgy is preferred where the highest purity and selective recovery are most important; whereas hybrid methods are most effective when combining resistance to material heterogeneity with complete metal recovery—including lithium—is required [40,83,88].

### 2.6. Justification for the Need to Develop Direct Recycling

Direct recycling techniques focus on physically separating active materials from battery electrodes and directly restoring their structure and composition. Lü et al. explain that this method involves repairing defects in active materials through processes such as lithium replenishment and heat treatment, allowing the materials to be reused without significant deterioration in their properties [92]. This approach contrasts with traditional methods, in which high-temperature processing often leads to a loss of integrity of the active materials, making them less suitable for reuse [26]. By preserving the functionality of these materials, direct recycling can significantly increase the overall recovery rate of key battery components.

Conventional recycling processes, in particular pyrometallurgy and hydrometallurgy, are associated with high energy consumption and significant carbon dioxide emissions. Traditional pyrometallurgical methods can cause high greenhouse gas emissions due to the energy-intensive nature of smelting processes [26]. Direct recycling methods, however, aim to operate at lower temperatures and with lower energy consumption, potentially resulting in a smaller carbon footprint. Filomeno and Feraco emphasize that the development of direct recycling techniques is essential to minimize the environmental impact of energy-intensive recycling practices [26].

Direct recycling processes can simplify the recycling process by reducing the number of complex steps required in traditional methods, such as leaching and precipitation, which often generate hazardous waste and require intensive processing [93]. The single-vessel regeneration methods discussed by Shang et al. illustrate how these direct techniques can streamline the process, making it less labor-intensive and more cost-effective [94]. Simplifying recycling processes may encourage greater industry involvement and facilitate the development of recycling infrastructure [95].

As demand for sustainable practices grows, governments around the world are beginning to establish regulatory frameworks that mandate recycling and the use of recycled materials in battery manufacturing. The trend toward requiring minimum recycled content is expected to intensify, as is the recognition of the need for sustainable end-of-life battery management [96]. Developing efficient direct recycling methods will help manufacturers meet these requirements, ensuring compliance while leveraging the strategic value of recycled materials.

The growing demand for lithium-ion batteries in electric vehicles and renewable energy systems is exacerbating the problem of resource scarcity, particularly for key elements such as lithium, cobalt, and nickel [97]. The development of direct recycling processes can significantly reduce dependence on primary raw materials by enabling the reuse of existing materials, thereby promoting a circular economy [96]. Meegoda et al. emphasize that recycling used batteries leads to a significantly lower environmental impact than producing new units from raw materials, highlighting the importance of developing effective recycling strategies in light of growing demand [98].

The development of direct battery recycling methods is essential due to their potential for effective recovery of active materials, a reduction in carbon footprint, simplification of recycling processes, compliance with regulatory standards, and addressing resource scarcity. With the growing emphasis on sustainability in battery technology, there is an urgent need to adopt and optimize direct recycling processes to create a more resilient and environmentally friendly battery economy.

## 3. Direct Recycling in Lithium-Ion Batteries

Direct battery recycling refers to innovative processes aimed at restoring the functionality of used lithium-ion battery materials, namely their active cathode components, without the need for destructive processing methods often found in traditional recycling methods. The main goal of direct recycling is to renew and regenerate degraded cathodes, thus enabling their direct reuse in new battery systems, which effectively contributes to a circular economy in battery production.

In the context of lithium-ion batteries, direct recycling involves methods that allow for the recovery and revitalization of active materials such as nickel-cobalt-manganese oxide (NCM) and lithium iron phosphate (LFP) through techniques that preserve their structural integrity. These processes typically involve selective leaching, surface regeneration, and lithium ion replenishment to restore the original electrochemical properties of cathode materials while minimizing structural degradation. For example, Jia et al. emphasize that direct recycling focuses on removing defects in spent LiFePO_4_ (LFP) cathodes that affect their performance by applying chemical treatment that regenerates the materials without the need for intensive processing [99].

The goal of developing direct battery recycling methods is multifaceted. Direct recycling offers the potential for higher recovery rates of valuable materials compared to traditional recycling methods, which can cause structural damage to active materials. Gao et al. point out that direct regeneration enables the creation of a closed-loop system in which processed active materials undergo minimal degradation, maintaining high performance for reuse in new batteries [100].

The direct recycling process is designed to operate at lower energy requirements, thereby minimizing the greenhouse gas emissions associated with traditional recycling. It aims to reduce the significant carbon footprint of pyrometallurgical and hydrometallurgical methods, thereby promoting a more sustainable approach to battery waste management. However, while some studies indicate a reduction in emissions, others suggest that the overall environmental impact may vary depending on the specific recycling methods and technologies used [76].

Traditional recycling methods often focus on dismantling battery components to recover base metals, potentially neglecting the inherent value and distinctive properties of battery-grade materials. Direct recycling, on the other hand, aims to preserve the complex structures found in cathodes, allowing desirable properties such as high capacity and cycle stability to be retained. This approach helps alleviate pressure related to resource depletion [101,102].

The economic benefits of direct recycling stem from lower operating costs associated with energy and material consumption. Processes such as cathode recycling, as explained by Yang et al., provide cost-effective ways to revitalize used materials for the production of new batteries, which translates into more accessible battery technologies for manufacturers [89]. In addition, increased efficiency can lead to reduced demand for new sources of lithium, which ultimately stabilizes market prices [97].

With the growing number of regulations on waste management and material recovery, direct recycling is in line with the drive for cleaner and more sustainable manufacturing practices. As battery manufacturers and policymakers emphasize closed-loop systems, direct recycling is becoming a proactive solution for meeting regulatory requirements while fostering innovation in recycling technology [103,104].

Direct battery recycling is a key approach in the evolution of lithium-ion battery management. By focusing on the preservation and regeneration of cathode materials, direct recycling not only increases material recovery efficiency but also reduces environmental impact and aligns with the growing emphasis on sustainability in the battery industry. As this field develops, it will play a key role in closing the battery material loop and supporting a truly circular economy.

**Figure 6:**
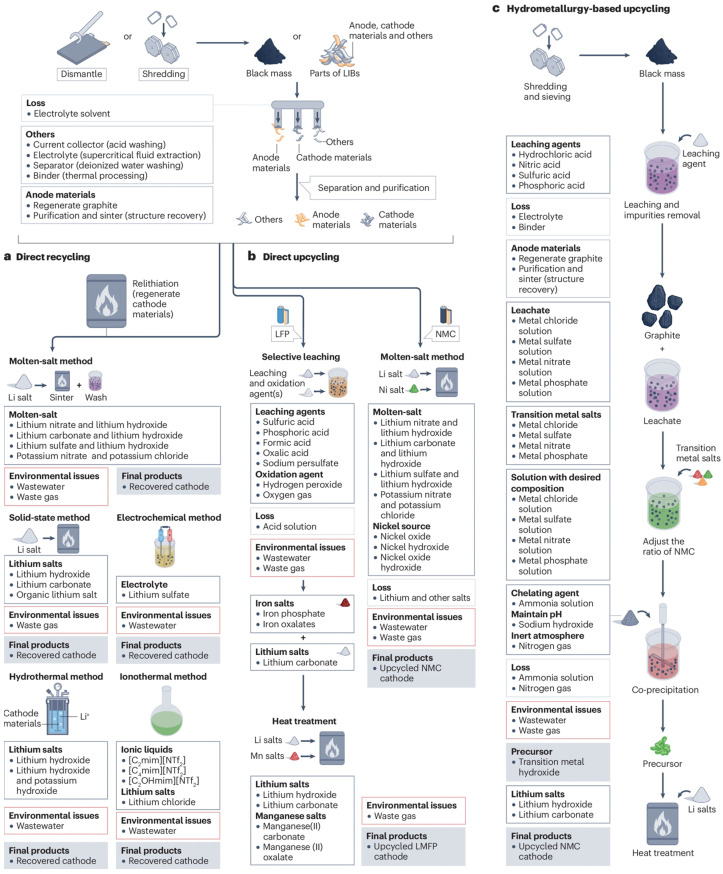
Diagram of the direct battery recycling process, showing the main stages of recovery and regeneration of active materials [105].

Figure 6 shows a diagram of the direct recycling process, illustrating the main stages of recovering active materials from batteries, starting with cell dismantling, through component separation and processing, to the regeneration of materials ready for reuse. This diagram provides an understanding of both the structure and sequence of technological operations used in direct battery recycling, showing the links between the individual stages of the process and the key actions leading to the preservation of the properties of active materials.

### 3.1. Technological Process

The first stage of the direct recycling process involves dismantling used lithium-ion batteries to access their internal components. This stage begins with safely discharging the batteries to prevent hazards associated with residual electrical energy. Once discharged, the batteries are mechanically opened or cut open to expose their internal components, which typically include electrodes, separators, and electrolyte materials. To make this process efficient and effective, mechanical methods such as shredding or cutting are often used [78]. During disassembly, special attention must be paid to securing hazardous materials such as electrolytes and complying with environmental regulations on waste management [78].

After dismantling the battery, the next step is to separate the individual components. At this stage, the focus is primarily on isolating the cathode and anode materials, which are the most valuable in terms of resource recovery. Techniques such as screening, magnetic separation, and flotation can be used to effectively separate metal components from polymeric materials. As described by Li et al., effective separation of components is crucial because it allows for targeted recycling of active materials in electrodes while minimizing contamination [106]. For example, materials such as graphite used in the anode and lithium transition metal oxides from the cathode must be isolated to facilitate subsequent processes.

The regeneration of active materials is at the heart of the direct recycling process. This stage involves restoring the electrochemical functionality of cathode and anode materials without altering their structural integrity. Methods such as chemical reduction and solid-state synthesis are commonly used. For example, in the case of lithium cobalt oxide (LiCoO_2_), recycling may involve treating the spent material with lithium salts to replenish the lithium content while simultaneously applying thermal treatment to restore the crystal structure and performance [107]. The regeneration process should also focus on removing contaminants generated during battery degradation, as these can adversely affect the performance of the regenerated materials. The ability to maintain or restore the functionality of the processed materials is essential, as it determines the cost-effectiveness of the direct recycling method in future applications.

The final stage is the regeneration of electrodes using regenerated active materials. This stage involves recombining the recovered cathode and anode materials with appropriate conductive additives and binders to form composite electrode structures. According to Wu et al., regeneration requires careful control of the composition and homogeneity of active materials to ensure optimal performance in new battery systems [108]. The formed electrodes can then be assembled into new battery cells, ready for use in applications ranging from electric vehicles to portable electronics. This step underscores the goal of creating a closed-loop system in which used materials are reused to produce new batteries, significantly reducing the demand for virgin raw materials and the associated environmental impact [109].

Direct battery recycling is a promising solution that addresses many of the challenges associated with traditional battery recycling methods. Encompassing the stages of disassembly, separation, regeneration, and remanufacturing, this process aims to increase recovery rates while maintaining material integrity and reducing environmental impact. Continued advances in technology and methodology in this field will be critical to shaping a sustainable future for energy storage systems.

### 3.2. Advantages of Direct Recycling

Direct recycling processes significantly reduce energy and chemical consumption compared to traditional hydrometallurgical and pyrometallurgical methods. Conventional methods often require high temperatures and numerous chemical treatments, leading to increased carbon dioxide emissions and operating costs. As Xu et al. note, direct recycling avoids the need for such energy-intensive processes, allowing active materials to be recovered without intensive heating or chemical decomposition [82]. This efficiency not only minimizes energy requirements but also reduces the overall environmental impact of battery recycling. Studies have shown that by preserving the structural integrity of materials during direct recycling, energy consumption can be significantly reduced, potentially leading to lower greenhouse gas emissions compared to conventional methods [110,111].

One of the key advantages of direct recycling is that it preserves the structure and electrochemical properties of active materials. Traditional recycling methods often cause degradation of these materials, making them unsuitable for direct reuse in new batteries. For example, as pointed out by Hayagan et al., direct recycling enables the recovery and regeneration of cathode materials while maintaining their original properties [112]. This feature is essential for maintaining battery performance and safety, as it enables the production of high-quality recycled materials that can be reincorporated into the battery manufacturing process with similar performance characteristics to new materials [112,113].

Direct recycling supports the principles of the circular economy by promoting the sustainable development of battery materials. Instead of following a linear model in which materials go through extraction, use, and disposal, direct recycling facilitates the continuous reuse of battery components, thereby reducing waste and decreasing the demand for virgin raw materials [111]. Incorporating regenerative processes into direct recycling can help establish a sustainable life cycle for lithium-ion batteries, which is consistent with global sustainability goals and ensures a more secure supply chain for critical materials. As documented by Harper et al., this paradigm shift highlights the importance of closed-loop systems in improving the overall sustainability of battery-related industries [111].

Furthermore, with the growing demand for electric vehicles and energy storage solutions, the ability to effectively recycle and regenerate resources will be essential to meet market needs while addressing environmental concerns. Direct recycling methods provide a way to recover valuable metals and materials while limiting the environmental degradation caused by the extraction and processing of raw materials [76,114].

Overall, direct recycling of lithium-ion batteries offers significant benefits, including reduced energy and chemical consumption, preservation of valuable material properties, and facilitation of a circular economy. As battery recycling evolves in response to growing market demands and sustainability needs, direct recycling will play a key role in shaping the future of battery management and resource utilization.

### 3.3. Limitations and Challenges

One of the main challenges associated with direct recycling is the variability in the chemical composition of batteries in different lithium-ion formulations. Each battery may use different cathode chemical components, such as lithium nickel cobalt manganese oxide (NCM), lithium iron phosphate (LFP), and lithium cobalt oxide (LCO), which exhibit different properties and degradation mechanisms. This variability complicates the direct recycling process, as methods optimized for one chemical composition may not be universally applicable to others. For example, the efficiency of direct recycling processes can vary significantly depending on the composition of the electrodes and the specific degradation products. The development of standard recycling protocols that can accommodate a variety of battery chemistries remains a key obstacle to the implementation of direct recycling methods [106].

Another significant challenge facing direct recycling technologies is achieving a high level of purity in the recovered materials. Direct recycling often involves the physical recovery of electrical materials without extensive chemical decomposition; however, contaminants from additives, binders (such as polyvinylidene fluoride, PVDF), and other components can affect the purity of the final recycled products. The adhesive strength between cathode materials and aluminum current collectors, along with the properties of PVDF binder, hinders effective separation, leading to contamination. High purity is essential for reclaimed materials to ensure that they meet performance standards in new battery applications. Technologies that do not produce high-quality materials may face limited market acceptance and profitability [115].

Scaling direct recycling methods from laboratory to industrial levels poses a significant challenge. Current research shows promising results under controlled laboratory conditions, but industrial implementation may encounter difficulties related to cost, efficiency, and process optimization. Direct recycling requires established infrastructure to meet the unique demands of large-scale battery sorting, separation, and regeneration processes. The cost of developing and maintaining the necessary facilities can pose a significant financial challenge, especially in the early stages of technology deployment. Therefore, a key challenge is to find economically viable solutions that minimize initial capital expenditure while achieving sufficient scale [116].

The development of direct recycling technologies for lithium-ion batteries faces a number of limitations and challenges that must be addressed in order to increase their effectiveness and sustainability. Sensitivity to different battery chemistries, difficulties in achieving material purity, and issues related to scaling and costs are major obstacles to the widespread adoption of these methods. To meet these challenges, further research and innovation are crucial to improve direct recycling technologies, optimize processes, and design systems that can effectively adapt to the diverse chemical composition of lithium-ion batteries.

Table 3 presents a comparison of the three main metal recycling processes: pyrometallurgy, hydrometallurgy, and direct recycling, in terms of complexity, waste generation, energy consumption, and environmental performance, referred to as the “green score.” The complexity of the process reflects its degree of technological difficulty, with a higher value indicating a more complex process. Waste generation indicates the amount of waste produced during recycling, and energy consumption determines how much energy is needed to carry out the process. The green score is a summary assessment of the process’s environmental impact, where a higher number indicates a greater negative impact. Pyrometallurgy is the most complex process, with a complexity score of 8, but compared to the other processes, it has relatively low energy consumption (5) and moderate waste generation (6), which translates into the lowest green score of 19. This means that, under laboratory conditions, it is a relatively environmentally friendly process, despite its technological difficulty. Hydrometallurgy is slightly less complex (6), but generates more waste (7) and requires more energy (7), resulting in a green score of 20, placing it in the middle in terms of environmental friendliness. Direct recycling is moderately complex (7), but at the same time has the highest energy consumption (8) and the largest amount of waste (8), resulting in a green score of 23, which is the least favorable from an environmental point of view. On a laboratory scale, pyrometallurgy is the most environmentally efficient process, hydrometallurgy is a compromise between process simplicity and environmental impact, while direct recycling, despite its relatively moderate complexity, generates the highest energy and waste loads [18].

Table 4 compares different strategies for processing end-of-life lithium-ion batteries (EoL LiBs) along with their associated unit costs in USD per ton and detailed comments on the specifics of each method. The first approach, referred to as refurbishing/repurposing, involves refurbishing or reusing batteries. In the case analyzed, it was assumed that the original battery (BOL—Beginning of Life) has a capacity of 115 Wh/kg, while the refurbished battery operates at 50–60% of the depth of discharge (DOD) relative to its initial state. Processing costs vary significantly depending on the level of processing, which includes cells, modules, or entire battery packs, and range from $2013 to $7452 per ton. The second method is direct recycling, which involves the direct recovery of materials, with the cathode accounting for approximately 25% of the cell’s weight. The cost of this process is $4447/ton. Another category is pyrometallurgy, which includes both a direct approach, in which mechanically unprocessed batteries are fed directly into the furnace, and a multi-stage approach, in which batteries are first mechanically prepared before being fed into the furnace. The cost range for this method is much wider, ranging from $26 to $1851/ton. The last strategy analyzed is hydrometallurgy, which includes the costs of mechanical preparation of batteries, with the base material (BM) share being 40% or 50% of the weight of the used battery. For a 40% BM share, the costs range from $1555 to $4819/ton, while for a 50% BM share, the costs range from $1582 to $5662/ton. The tabular analysis shows that the costs of processing EoL LiBs vary greatly, depending on the recycling strategy adopted, the degree of mechanical preparation, and the type of components processed. Reuse and hydrometallurgical methods have a higher unit cost compared to pyrometallurgy and direct recycling, reflecting the complexity of the processes and the potential benefits of recovering high-value materials.

## 4. Latest Developments and Entities Operating in the Field of Direct Recycling of LIBs

One of the most recognizable and priority programs/projects related to LIB recycling, including direct recycling, is the ReLiB project, coordinated by the University of Birmingham as part of the Faraday Institution. The consortium consists of leading British universities (including Edinburgh, Newcastle, Leicester, Oxford, Imperial College London, and UCL). The aim is to provide technological solutions and expertise for the reuse and full recycling of Li-ion batteries from the automotive sector and beyond. The project focuses on “short loop” recovery and low-impact separation and regeneration pathways for cathode and anode materials to reduce the cost, energy consumption, and environmental footprint of recycling while improving the safety and productivity of battery waste (especially from EVs) dismantling/processing. The project has resulted in an extensive bibliography of publications on direct recycling methods, and the team has also received industry awards [117,118,119,120,121,122,123,124].

Another example is BATRAW, a four-year EU project whose main objective is to develop and demonstrate two innovative pilot lines for the sustainable management of end-of-life Li-ion batteries (EVs, domestic batteries, production waste). The scope covers the entire end-to-end chain: semi-automatic dismantling, assessment of second-life/repair possibilities, recycling and recovery of cathode/anode materials to industrial quality; the project is coordinated by a consortium involving BeePlanet, LEITAT, Orano and other research and industrial partners [125].

Another example is the ReCell Center, the first R&D center in the US dedicated to Li-ion battery recycling, established by the US DOE (Vehicle Technologies Office) and run by Argonne National Laboratory with the participation of national laboratories and universities [126]. Mission: to reduce the cost and energy consumption of recycling and increase the value of recovered materials in order to build a national, cost-effective battery recycling chain (EV, electronics, energy storage). ReCell conducts advanced research in direct recycling, and detailed achievements and the research results are described in scientific publications such as [82,104,127,128,129,130,131,132,133].

Table 5 presents selected initiatives, projects, and research units involved in the development of direct recycling technologies for lithium-ion batteries (LiB), covering both scientific and industrial components. The list includes various types of organizations: research consortia (e.g., ReCell Center), start-ups and industrial pilots (Princeton NuEnergy), small and medium-sized technology companies (OnTo Technology), national programs (ReLiB—Faraday Institution), academic groups (University of Leicester), as well as projects funded under European initiatives (RecyLIB, BATRAW, REVITALISE) [125,126,134,135,136,137,138,139]. Geographically, most activities are concentrated in the United States (ReCell Center, Princeton NuEnergy, OnTo Technology) and Western Europe (UK, Germany, Spain, Sweden), which indicates the leading role of these regions in research on sustainable battery recycling [126,134,135]. Initiatives in the US are characterized by a strong focus on the development of industrial and pilot technologies, while in Europe, there is a strong emphasis on research and development projects, co-financed by European programs, covering both technological and environmental aspects. The scope of research and development in the table focuses mainly on so-called “direct recycling,”, i.e., the direct regeneration of cathode materials, including the recovery, reconditioning, and reintegration of active battery components. Some projects develop innovative approaches, such as low-temperature regeneration using plasma (Princeton NuEnergy) or “nanomelusions” in a short recycling cycle (University of Leicester) [134]. In addition, some projects integrate aspects of sustainable development, such as CO_2_ emission reduction (OnTo Technology) or green recycling in the context of LiB and Na-ion batteries (REVITALISE) [139]. The overview shows the growing diversity of technological and organizational approaches in the field of direct battery recycling, combining basic research, industrial pilot projects, and European projects, with a strong focus on both material efficiency and environmental aspects.

## 5. Key Challenges and Forecasts for Direct Recycling

One of the biggest challenges associated with direct recycling is the sensitivity of the recycling process to different battery chemistries. Lithium-ion batteries use different cathode materials, including nickel-manganese-cobalt oxide (NCM), lithium iron phosphate (LFP), and lithium cobalt oxide (LCO), each of which has unique properties and degradation mechanisms [106]. As Li et al. point out, the diversity of battery compositions requires the development of tailored recycling processes, as technologies that are effective for one chemical composition may not be suitable for another [106]. This specificity may hinder the scalability of direct recycling, as manufacturers must consider a range of materials that may require processing.

Achieving a high level of purity in recovered materials is a significant obstacle to direct recycling. Contaminants from binders, additives, and residues can negatively affect the quality of reclaimed materials [140]. Studies have shown that in order to meet the stringent performance standards for new battery cells, the purity of recovered materials must generally exceed certain thresholds, often greater than 99% [112]. Hayagan et al. note that without effective separation methods, ensuring the necessary level of purity after recycling can significantly limit the use of recycled materials in the production of new batteries [112]. Therefore, improving separation technologies and processes to minimize contamination remains a key area of interest in the context of improving direct recycling performance.

The implementation of direct recycling technology on an industrial scale poses significant challenges. Although laboratory-scale experiments show potential, transferring these processes to an industrial scale requires solving several issues, including cost, efficiency, and safety. Direct recycling requires specialized facilities capable of handling the diverse chemicals and complex structures found in lithium-ion batteries, which entails significant upfront investments [141]. Furthermore, achieving economic viability is essential, especially when competing with traditional methods, which may have lower initial costs but a greater long-term environmental impact [142]. Therefore, optimizing production efficiency and reducing costs will be critical to the commercial success of direct recycling technologies.

As demand for lithium-ion batteries grows, the importance of sustainable recycling solutions is likely to increase. Several predictions can be made about the future development of direct recycling methods. Further research and development is expected to yield innovative direct recycling technologies that will improve efficiency and effectiveness, enabling the processing of a wider range of batteries with less variability in the results. Innovations such as selective chemical treatments and advanced material separation techniques are likely to increase the scalability and applicability of direct recycling [143]. The future regulatory framework is expected to support direct recycling initiatives. Policies promoting circular economy principles and encouraging sustainable practices in battery production and disposal can provide funding and support for the development of direct recycling technologies. For example, new regulations in regions such as the European Union and the United States may require increased recycling rates and recycled content in the manufacture of new batteries, which will catalyze innovation in this area [144]. As a more integrated approach to resource management gains traction, direct recycling is expected to play a key role in establishing a circular economy for lithium-ion batteries. The emphasis on material reuse and regeneration is likely to drive demand for technologies and practices that prioritize minimal waste generation, efficient resource recovery, and sustainable battery system design [76].

Although direct recycling faces challenges related to chemical composition sensitivity, material purity, and economic viability, continuous progress and favorable policy frameworks suggest a bright future. If these obstacles can be satisfactorily overcome, direct recycling could make a significant contribution to achieving sustainable development goals in the context of batteries.

## 6. Digitization Techniques Used in Direct Recycling Processes of Lithium-Ion Batteries

Numerical modeling in many fields can help reduce experimental work, accelerate device design, and optimization. Various digitalization techniques are also useful for the entire life cycle of a battery, from production through operation to end of life and its recycling [145]. The first step in the direct recycling process of lithium-ion batteries, which involves dismantling them, is difficult to automate due to the variety of sizes and shapes. Additionally, they contain hazardous and flammable materials, which poses a serious threat to human health. A platform for automatic battery dismantling is proposed in work by Lu et al. [146]. It consists of a battery classification module that determines its size, a cutting module with temperature monitoring, and a module for assessing cut quality. A convolutional neural network is used to select the correct battery from over hundreds of different types based on size, shape and brand model. The Bayesian Optimization is applied to find the learning rate, number of epochs, batch size and depth of the network. The network learns, in offline mode, the most representative feature set for classification. Once training is complete, the network can perform online image classification at approximately 30 frames per second on a single graphics processing unit. Information about the battery dimensions is then passed to the cutting module, which uses it for its positioning. Based on previously conducted experimental studies, a model is built that predicts cutting temperature behavior. Information from the predictive model and the installed thermal camera allows the optimal cutting speed to be determined online, taking into account the temperature and cutting time. The last control module also uses a neural network to detect and mitigate cutting defects. A study by Redmon et al. [147] demonstrates the use of the single-stage deep learning architecture YOLOv2 for battery device classification, while the work of Sterkens et al. [148] applies this approach to battery detection and battery structure recognition. This work demonstrates the effectiveness of deep learning for object detection in X-ray transmission generated images for both automatic battery removal and sorting.

Numerical methods are also used to develop ways to separate individual battery components. A computer simulation of a cyclone used to separate lithium iron phosphate particles from lithium battery waste materials is presented in a paper by Pang et al. [149]. The gas phase is assumed to be incompressible and its flow to be turbulent. The Reynolds stress model is used to solve the system of he Reynolds-averaged Navier–Stokes equation [150]. The Euler-Lagrange method is used to model the dispersed phases [151]. After 15 separations, approximately 76% of the LFP particles are separated in the entire feed material. For aqueous dispersion of cathode materials such as LFP, a tubular centrifuge can be used [152]. To derive the desired material parameters from laboratory tests, a tailored optimization procedure is designed using the genetic algorithm.

A key step in the direct recycling process is the regeneration of active materials such as the anode and cathode. Anode degradation in lithium-ion batteries is associated with the growth of the solid electrolyte interphase, which in subsequent operating cycles consumes lithium ions, increases internal resistance and limits ion exchange. Additionally, cracking and mechanical degradation of graphite also occur. In the cathode, the crystal structure responsible for the insertion and removal of lithium ions (intercalation and deintercalation) degrades. Lithium reacts with the cathode electrolyte interphase surface layer, and oxygen atoms are released.

A publication by Kim et al. describes the use of redox mediators for efficient direct recycling by rapidly transferring charges between the cathode and the Li metal and providing Li+ ions for re-lithiation of degraded cathode materials. For this purpose, 3,5-di-tert-butyl-o-benzoquinone is used. Structural changes in the used cathode materials are studied using in situ spectroscopy and density functional theory calculations [102].

A direct recycling process involving hydrothermal re-lithiation to restore the lithium stoichiometry followed by sintering to restore the microstructure was described in work by Lai et al. [153]. Hydrothermal relithiazation is modeled using adsorption kinetic models and adsorption isotherm models. The implemented models showed that the hydrothermal relithiazation reaction is dominated by chemisorption, and the hydrothermal relithiazation reaction is controlled primarily by monolayer adsorption. The calculated thermodynamic parameters indicate that the hydrothermal relithiazation reaction is an endothermic and nonspontaneous process.

A protocol for electrochemical re-lithiation of lithium half-cells was developed, enabling rapid regeneration of their cathodes from decommissioned cells to their original state. The Doyle-Fuller-Newman model is used to determine the half-cell discharge performance characteristics. Lithium conservation equations are written for the active solid particles and electrolyte phase. The Butler-Volmer kinetics with symmetric charge transfer coefficients describe the lithium intercalation reaction at the cathode [154].

Anode active materials from spent lithium–ion batteries can also be recovered and potentially used in new batteries. Froth flotation kinetics are simulated using computational fluid dynamics and surface chemistry in a publication on that subject [155]. The simulation results are used to generate data for training a deep learning neural network. This network then predicts the flotation of the battery’s active material. The analyses performed confirm that particle diameters < 5 μm have a major impact on cell performance and that an additional process is necessary to improve the quality of the recovered anode materials. A review of the available literature shows that there are few works devoted to the development of methods for modeling the direct recycling process of lithium-ion batteries. Therefore, significant development of modeling methods for this process should be expected [155].

## 7. Conclusions

The growth rate of global production and demand for lithium-ion batteries (LIBs) exceeds the current capacity for end-of-life battery management, which poses significant challenges in terms of the circular economy and raw material security. With the growing scale of LIB applications in electromobility, renewable energy storage, and portable electronics, a sharp increase in the number of used cells is predicted over the next two decades. Due to the inevitable degradation of active materials—resulting from processes such as lithium ion deintercalation, crystal structure decay, and the accumulation of by-products in the electrolyte—these batteries require controlled and safe processing at the end of their life cycle.

The pyrometallurgical and hydrometallurgical technologies that have dominated to date, although effective in recovering valuable metals (Ni, Co, Li, Mn), are characterized by high energy consumption, chemical reagent consumption, and a significant environmental footprint, with a simultaneous loss of the structure of active cathode phases. In response to these limitations, there is growing interest in the concept of direct recycling, which aims to preserve the morphological and chemical integrity of cathode materials and regenerate them through relithiation or rejuvenation processes. This approach minimizes the loss of embedded energy and reduces the number of processing steps required to achieve battery-grade material quality.

From a technical point of view, direct recycling involves a number of innovative methods, such as low-temperature chemical regeneration, hydrothermal treatment, gas phase reactions, plasma surface activation, and the use of nano-milk solutions and deep eutectic solvents (DES). This strategy is part of the “green chemistry” trend, which aims to reduce the consumption of primary resources and toxic substances while increasing the material efficiency of the entire battery life cycle.

The scale of current research, European projects (e.g., RecyLIB, BA-TRAW, REVITALISE) and industrial initiatives (ReCell Center, Princeton NuE-nergy, OnTo Technology) suggests that the share of direct recycling in the global battery material recovery market may grow rapidly in the coming decade. Nevertheless, its widespread implementation still faces numerous barriers of technical (including waste heterogeneity, cathode degradation, chemical composition control), logistical (lack of standardization of collection and sorting systems), quality (maintaining electrochemical parameters after regeneration), and regulatory natures (lack of a consistent legal framework and recycling standards on an international scale).

Therefore, further research and development should focus on developing innovative, sustainable, and economically viable recycling technologies that enable the recovery of high-purity materials with minimal environmental impact. The development of such technologies is crucial not only for reducing hazardous waste and greenhouse gas emissions, but also for Europe’s raw material security and the implementation of the European Green Deal strategy. In the long term, the implementation of direct recycling is an indispensable element of the transition towards a fully closed life cycle for lithium-ion batteries, combining technological, environmental, and economic aspects [155]. In the long term, the implementation of direct recycling is an indispensable element of the transition towards a fully closed life cycle for lithium-ion batteries, combining technological, environmental, and economic aspects [156].

## Figures and Tables

**Table 1:** Advantages and disadvantages of pyrometallurgical recycling of lithium-ion batteries (LIB)—examples of industrial companies applying it in practice [30,33,48,49,50,51,52,53,54,55,56].

Aspect	Characteristics
Advantages	Technological simplicity and high level of process maturity The ability to process a mixture of different types of cells without the need for prior sorting Good industrial scalability and the possibility of integration with existing metallurgical installations
Disadvantages	High energy consumption due to the high-temperature nature of the process Gas emissions (HF, CO_2_, SO_2_) and the risk of atmospheric pollution Loss of lithium and aluminum in slag, limiting overall recovery efficiency Degradation of cathode materials and inability to reuse them directly
Examples of technologies	Umicore, Glencore, Sumitomo Metal Mining

**Table 2:** Advantages and disadvantages of hydrometallurgical recycling of lithium-ion batteries (LIB) and examples of industrial companies applying it in practice [33,48,49,53,66,67,68,69,70,71,72].

Aspect	Characteristics
Advantages	High metal recovery efficiency (>90%) Lower energy consumption compared to pyrometallurgical methods Possibility of recovering lithium in the form of salts (e.g., Li_2_CO_3_, LiOH)
Disadvantages	The need to use strong chemical reagents (H_2_SO_4_, HCl, H_2_O_2_) Generation of large amounts of wastewater and waste requiring neutralization Process complexity, involving numerous stages of extraction and purification Lack of preservation of the cathode material structure, which prevents its direct reuse
Examples of technologies	Retriev Technologies, OnTo Technology, Fortum, Toxco

**Table 3:** Comparison of metal recycling processes in terms of complexity, waste generation, energy consumption, and environmental impact [18].

Process	Complexity	Waste Generation	Power Consumption	Green Score
Pyrometallurgy	8	6	5	19
Hydrometallurgy	6	7	7	20
Direct recycling	7	8	8	23

**Table 4:** Comparison of costs and characteristics of selected methods for processing end-of-life lithium-ion batteries (EoL LiBs), including refurbishment/reuse, direct recycling, pyrometallurgy, and hydrometallurgy, taking into account the mass fraction of the base material and the depth of energy discharge [18].

Processing Approach	Notes	Cost (USD/Tonne of EoL LiBs)
Refurbishing/repurposing	Based on 115 Wh/kg battery at BOL, and the refurbished LiB has 50–60% DOD relative to at BOL. Cost variance considering cell, module or battery treatment.	2013–7452
Direct recycling	Cathode material makes up 25% of the cell by mass.	4447
Pyrometallurgy	Direct—mechanically untreated LiBs directly fed into furnace. Multi-step—mechanical LiB pretreatment steps before charging in furnace.	26–1851
Hydrometallurgy	Contains cost of mechanical treatment with BM making up 40% of the EoL LiB.	1555–4819
	Contains cost of mechanical treatment with BM making up 50% of the EoL LiB.	1582–5662

**Table 5:** Selected initiatives and projects in the field of direct recycling of lithium-ion batteries [125,126,134,135,136,137,138,139].

Name	Type	Focus	city	Country	Lat	Lon	Source
ReCell Center (DOE)	Research consortium	Direct recycling R&D (component recovery & relithiation)	Lemont (Chicago area)	USA	41.713	−87.981	ReCell Center–Direct Recycling of Materials page
Princeton NuEnergy (PNE)	Startup/industrial pilot	Low-temperature plasma-assisted direct regeneration of CAM	Princeton	USA	40.357	−74.667	PNE technologies page + Princeton news
OnTo Technology	SME/technology developer	Direct recycling, cathode healing, CO_2_ inertization	Bend, OR	USA	44.058	−121.315	OnTo Technology site & NAATBatt PDF
ReLiB (Faraday Institution)	National research programme	Battery recycling incl. direct routes & upcycling	Birmingham	UK	52.45	−1.93	ReLiB & Faraday Institution pages
University of Leicester–Direct recycling group	Academic group	Short-loop direct recycling (nanomelusions), LFP relithiation	Leicester	UK	52.636	−1.139	Leicester pages & 2023 RSC paper
RecyLIB (Fraunhofer ISC)	EU project	Direct recycling & reintegration of active materials	Würzburg	Germany	49.794	9.932	RecyLIB site & factsheet
BATRAW (LEITAT)	EU project	End-of-life packs; developing routes incl. direct reuse of	Terrassa (Barcelona)	Spain	41.563	2.008	BATRAW project site/CORDIS
REVITALISE (BATTERY2030+)	EU research project	Green recycling incl. direct approaches for LiB/SIB	Gothenburg	Sweden	57.689	11.974	BATTERY2030+ REVITALISE

## Data Availability

No new data were created or analyzed in this study. Data sharing is not applicable to this article.
